# IMPACT OF PEDIATRICIAN TRAINING ON DIAGNOSIS OF DEVELOPMENTAL HIP DYSPLASIA

**DOI:** 10.1590/1413-785220253302e285336

**Published:** 2025-06-02

**Authors:** Nilton Orlando, Claudio Santili

**Affiliations:** 1 Universidade do Estado do Amazonas (UEA), Faculdade de Medicina, Escola de Saúde, Amazonas, AM, Brazil.; 2 Santa Casa de Misericórdia de Sao Paulo, Faculdade de Ciências Médicas, Departamento de Ortopedia e Traumatologia, Sao Paulo, SP, Brazil.

**Keywords:** Developmental Dysplasia of the Hip, Teaching, Diagnosis, Displasia do desenvolvimento do quadril, Ensino, Diagnóstico

## Abstract

**Objective:**

This research aims to promote the development of diagnosis and training of pediatricians using the simulated model "baby hips" and to evaluate the impact on the knowledge and skills of participants about the diagnosis.

**Method:**

An ecological study was employed using the tool the International Institute of Hip Dysplasia provided.

**Results:**

The World Health Organization considers developmental hip dysplasia (HD) a public health problem, and Brazil has no national policy for neonatal screening for HD. Delayed diagnosis impacts public resources with hospitalizations and surgeries, while early diagnosis promotes the opportunity for conservative treatment with good results and low cost. The pediatricians are essential for neonatal screening with Barlow and Ortolani tests, which have high specificity and sensitivity during this period of the child's life.

**Conclusions:**

A positive impact on diagnostic competence was obtained, indicating the need to promote training programs for pediatricians and stimulate public health authorities to include HD in the neonatal screening practiced in Brazil. ***Level of evidence II; Analytic observational cross over study.***

## RESUMO

**Objetivo:**

Esta pesquisa pretende promover o desenvolvimento diagnóstico e treinamento de médicos pediatras utilizando o modelo simulado "baby hips" e avaliar o impacto sobre o conhecimento e habilidades dos participantes a respeito do diagnóstico.

**Método:**

Foi empregado um estudo ecológico utilizando a ferramenta cedida pelo instituto internacional de displasia do quadril.

**Resultados:**

A Organização Mundial de Saúde considera a displasia do desenvolvimento do quadril (DDQ) um problema de saúde pública e o Brasil não possui política nacional para triagem neonatal interessando a DDQ. O atraso diagnóstico impacta negativamente os recursos públicos com internações e cirurgias enquanto o diagnóstico precoce promove a oportunidade do tratamento conservador que demonstra bons resultados e baixo custo. O pediatra é fundamental para a triagem neonatal com os testes de Barlow e Ortolani, que apresentam elevada especificidade e sensibilidade nesse período da vida da criança.

**Conclusão:**

Observou-se um impacto positivo sobre a competência diagnóstica o que indica a necessidade da promoção de programas de treinamento ao pediatra e reforça que as autoridades de saúde pública adicionem a DDQ na triagem neonatal praticada no Brasil. ***Nível de evidência II; Estudo observacional analítico cruzado.***

## INTRODUCTION

Hip instability is one of newborns’ most common alterations during routine physical examination, diagnosed by the Barlow and Ortolani tests.^1^

Described as a morphological alteration that ranges from an unstable joint to an installed dislocation, it is characterized by the anatomical anomaly between the femoral head and the acetabulum.^2^

The late diagnosis of HD directly implies a decrease in the child's quality of life, impacts on high health costs, and makes HD a public health problem, according to the World Health Organization, which determines the need for routine neonatal screening.^3^

The exact cause of this alteration is unknown, but several factors are involved, such as family history, female gender, oligohydramnios, twin babies, macrosomic babies, and intrauterine pelvic positioning, the latter standing out as the most important, increasing the incidence of HD.^4^

The number of new cases of HD in children without risk factors is estimated at 11.5/1,000 live births, based on meta-analysis and multiple logistic regression protocols, but this figure varies according to the geographical area studied. The relative risk in the case of a positive family history is 1.7 times higher (6.4/1,000 boys and 32/1,000 girls) and the pelvic presentation at birth, compared to the cephalic position, increases this risk by 6.3 times.^5,6^

When diagnosed early, conservative treatment for typical HD, i.e., without teratological changes, is effective, using suspenders and/or orthoses, and when late, the need for interventions increases the cost of treatment, increases morbidity, and has fewer effective results than conservative treatment.^7,8^ The American Academy of Pediatric Orthopaedics recommends evaluating all newborns with Ortolani and Barlow tests, which show high specificity and sensitivity up to the third month of age.^8^

There has been a lack of knowledge among health professionals in Brazil, especially pediatricians and pediatric residents responsible for clinical screening, resulting in late referral of patients for treatment.^9^

Continuous education and training of health professionals using the simulated *"baby hips* ^®^ *"* model show effective results in early diagnosis.^10^

Brazil currently has no neonatal screening strategy and the main instrument, the Caderneta da Criança, has neglected this care.^11^

This failure leads to more tests being requested and unnecessary medical referrals, increasing the costs of this disease and making the surgical treatment of HD in Brazil more frequent, with significant costs to the Unified Health System.^12,13^

This study evaluated the impact of training pediatricians on their knowledge and skills in diagnosing developmental dysplasia of the hip using a simulated *"baby hips* ^®^ *"* model*.*

## MATERIAL AND METHODS

A total of 312 pediatricians took part in this analytical, analytic observational cross over study. They received training in diagnosing HD using the simulated "*baby hips*" model and were assessed on their knowledge and skills in neonatal clinical diagnosis at two points in time, pre-and post-training.

**Figure 1:**
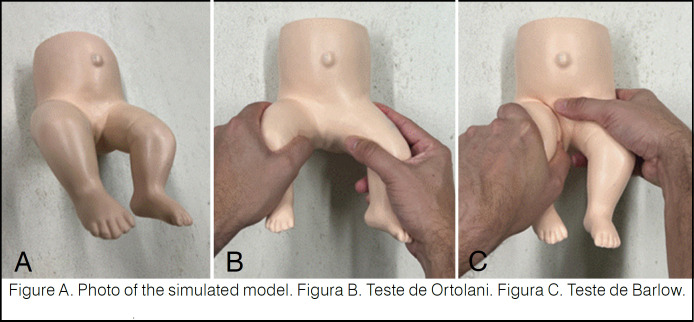
A simulated model was used for the physical examination of pediatric hips with developmental hip dysplasia.

Source: author's photos.

The pre-training assessment consisted of collecting data on the participants’ prior knowledge of HD and its early diagnosis by answering a questionnaire provided by the International Hip Dysplasia Institute using Google Forms^®^ and on their ability to perform the Barlow and Ortolani tests on a simulated "*baby hips* ^®^" model. (Figure 1)

The data collection questionnaire contained 18 questions, and the number of correct answers each participant provided classified them into three different groups: insufficient (zero to 5 correct answers), satisfactory (6 to 11 correct answers), and *good* (12 to 18 correct answers).

After this, a video with instructions for training in clinical diagnosis was shown, followed by a post-test containing the same questionnaire and simulated model applied previously.

The pre-and post-training scores were statistically analyzed with GraphPad Prism® software using the non-parametric data T-test, which was considered significant when *p < 0.05*.

For the evaluations relating to the 18-question questionnaire, the groups were divided into 3 groups, analyzed by the scatter plot, and these groups were called: *unsatisfactory* - between 0 and 5 hits; *fair* - between 6 and 11 hits and *good* - between 12 and 18 hits. The raw values analyzed the Ortolani and Barlow tests as right and wrong, and their representation in the percentage of participants analyzed.

All participants signed the informed consent form (ICF), and the research was approved by the ethics committee under C.A.A.E. No. 3462343.

## RESULTS

### About the collection instrument

The impact of pediatrician training on the diagnosis of hip dysplasia showed positive results with improvement in all categories in response to the questionnaire.

Those with unsatisfactory success rates fell from 26% to 9%. Those in the regular category improved from 13% to 35%, and those in the good category increased significantly applying the unpaired t-test, showing statistical significance with a *p-value of <0.05* between the values obtained pre- and post-training in all groups. (Table 1)

**Table 1:** The table shows the number of participants per group according to the number of correct answers to the questionnaire. The groups are unsatisfactory (0 to 5 hits), fair (6 to 11 hits), and good (12 to 18 hits).

Classification of the questionnaire evaluation by the number of correct answers	Number of hits of the participants pre-training	Number of correct answers post-training
Group Unsatisfactory hits 0 - 5	82	28
Group Regular 6 - 11 hits	189	174
Group Good Hits 12 - 18	41	110

The analysis of the overall score obtained in the questionnaire by the participants showed an increase in the average from 7.52 correct answers to 10.33 correct answers after the training, giving a higher concentration of participants classified as regular and percentages before and after, respectively from 41.7% to 57.3%, and a significant improvement in the group classified as good, containing 41 pediatricians pre-training, increased to 110 pediatricians post-training.

### About the Ortolani test

Analysis of the data on the Ortolani test performed by the participants was carried out using the ANOVA test and showed a significant difference between the variables before and after training, with a *p-value of <0.05*.

The number of errors decreased from 198 to 55 participants, while the number of correct answers increased from 114 participants before the training to 257 participants after the training, as shown in (Table 2).

**Table 2:** The table shows the number of participants and the percentage distribution on the Ortolani test. (*)

Simulated physical examination test	Distribution of participants with error pre-training	Distribution of participants with error post-training	Distribution of participants correctly pre-training	Distribution of participants correctly post-training
Ortolani	n (198) % (63)	n (55) % (18)	n (114) % (37)	n (257) % (82)

(*) all pre- and post-training conversion rate values showed statistical significance with a value of *p<0,05.*

### About the Barlow test

The Barlow test was the most unfamiliar among pediatricians, and 274 participants made mistakes. After training, the number of errors dropped to 150, and the number of correct answers rose from 38 to 162, as shown in Table 3. When treated by the ANOVA test, these data were statistically significant with a *p-value of <0.05*.

**Table 3:** The table shows the number of participants and the percentage distribution on the Barlow test. (*)

Simulated physical examination test	Distribution of participants with error pre-training	Distribution of participants with error post-training	Distribution of participants correctly pre-training	Distribution of participants with post-training success
Barlow	n (274) % (88)	n (150) % (48)	n (38) % (12)	n (162) % (52)

(*) all pre- and post-training conversion rate values showed statistical significance with a value of *p*<0.05.

## DISCUSSION

Developmental hip dysplasia (HD) affects 1% to 3% of newborns worldwide, is graded from hip instability to complete dislocation of the joint, and most often occurs in healthy children in isolation, masking the diagnosis.^14^

The ideal method for early diagnosis of HD has yet to be defined in several countries, ranging from clinical aspects to different and controversial radiological methods.^15^

However, it has been established that the physical examination is effective and universally accepted in the neonatal period through the Barlow and Ortolani tests, with specificity of up to 95%.^9^

According to the recommendations of the Pediatric Orthopaedic Society of North America (POSNA), the physical examination is fundamental and should be carried out first by the pediatrician. If positive, the patient is referred for an orthopedic evaluation to clarify or confirm the diagnosis of HD.

In Brazil, knowledge of HD among health professionals who perform newborn screening is poor, as 81% of professionals have never made an HD diagnosis. In this sense, mannequins can be useful for training students and healthcare staff.^13^

Therefore, continuing education and measures that are easy to apply and cost-effective could benefit many newborns.

This study carried out in Amazonas, provided pediatricians with theoretical and practical training on diagnosing developmental dysplasia of the hip using a simulated model and verified the impact of the training on the participants’ knowledge and skills.

We found that 86% of the participants had a fair or insufficient knowledge of HD prior to the training, as assessed by a questionnaire. The Ortolani and Barlow tests in a simulated model showed errors of 63% and 87%, respectively, indicating poor clinical diagnostic skills.

The study participants’ profiles showed low diagnostic capacity, with an average of 24% correctly diagnosing HD in the simulated model.

The positive impact on theoretical knowledge was seen post-training, with indices showing a significant improvement in the number of correct answers to the questionnaire, demonstrated by the increase in the good group.

The participants’ correct answers to the Barlow and Ortolani tests increased from 24% before training to 43% after training.

The data collection instrument used in this research was developed and supplied by the International Hip Dysplasia Institute and, like any other questionnaire-type data collection instrument, although it is a viable and pertinent technical tool to be used when dealing with problems whose objects of research correspond to empirical questions, involving the opinion, perception, position, and preferences of those surveyed, it can also be criticized when there are issues that compromise its construction and interpretation.^16,17^

Critically, the questionnaire drawn up by the International Hip Dysplasia Institute is difficult to interpret, with questions that could be improved by its authorial source to become a more accurate tool for assessing knowledge perception.

The participants’ responses to the data collection instrument averaged 7.5 and 10.3 correct answers before and after training, respectively.

Despite the increase in correct answers, it cannot be said that the data collection instrument is free from criticism and whether the data shows a gain in scores due to acquired learning or recent memory, since the data was collected in the same training session.

This research was carried out in Amazonas, where the distances between municipalities prevent their inhabitants from traveling quickly.

In this sense, telemedicine, regulated by the Federal Council of Medicine, can be used for continuing education on the diagnosis of HD, facilitating access to health information, and integrating regions of Brazil. Providing remote locations with contact with hospitals and specialized professionals regarding prevention, diagnosis, and health education has proved possible, making it possible to identify and track public health problems.^18^

The current situation in Brazil regarding neonatal screening for HD is flawed and does not provide the care that newborns need concerning the early diagnosis of HD.

In recent years, Brazil has made progress with public neonatal screening policies, such as the expanded search for the heel prick test offered by the SUS, including new rare disease screening groups, a high-cost measure that is very important for the health care of newborns.^19^

Despite current public health efforts in Brazil, simpler and less costly measures have been abandoned. The removal of hip assessment, which was available in previous editions of the child's booklet, demonstrates a step backwards in the care of newborns in Brazil.

The Ministry of Health (MoH) recommends the Ortolani test in the first two days of life and at subsequent childcare appointments. Ultrasound is recommended when the Ortolani test result is positive, a family history is present, the newborn has intrauterine positioning of the pelvic presentation type, or associated clinical manifestations such as congenital torticollis or foot malformations.^14^

Research has verified the need to improve pediatricians’ ability to diagnose HD, and technology tools should be employed to facilitate this. One example is the QR code with the acronym "Quick Response," which was developed by Masahiro Hara in 1994.^20^ Its application in the new editions of the Children's Booklet could provide information on the diagnosis of HD.

The training of pediatricians proved to be successful, with a positive impact on the practical context of the neonatal physical examination, increasing from 37% to 82% of participants being correct about the Ortolani test using the *"baby hips* ^®^ *"* model*.*

On the other hand, the Barlow test, which is less well known among pediatricians, was only 12% correct before training and reached 52% correct after training. This makes it clear that neonatal diagnosis can be improved with instruction and practical training for professionals who care for children in the neonatal period.

Screening, when carried out by pediatricians who suspect the diagnosis of HD, can lead to an increase in demand for specialized outpatient clinics. Once the diagnosis is confirmed, these clinics begin treatment appropriately, with a better outcome and lower costs for the public health system.

Many studies confirm the effectiveness of clinical screening alone in reducing late cases of HD. A simple and low-cost measure to implement and apply.^21^

Some countries, such as Australia, France, and the United Kingdom, have seen an increase in the number of children diagnosed late. This fact has been attributed to low knowledge of hip physical examination maneuvers before walking.^15^

In the UK, a competency program in neonatal clinical diagnosis has been launched to ensure that all doctors involved in the screening program receive training, periodic assessments, and adequate competencies to make the diagnosis of HD.^22,23^

In Brazil, Ordinance No. 1,130 of August 5, 2015, which establishes the national policy for comprehensive care for children (PNAISC) within the scope of the Unified Health System (SUS), in its article 7, deals with strategic actions of the axis of humanized and qualified care for pregnancy, childbirth, birth, and the newborn, referring, in paragraph VII, to neonatal screening. Therefore, we believe there are mechanisms already in place to update future editions of the children's booklet, improving the diagnosis of developmental dysplasia of the hip in newborns.

The authors, concerned about the current scenario due to the absence of a neonatal screening tool for HD in Brazil and understanding that the pediatrician is fundamental to the diagnostic search, point out that continued training is important and positively impacts diagnostic capacity.

At the same time, it is recommended that the Child Handbook be rectified, including a diagnostic field for the Barlow and Ortolani tests and the addition of a *QR code* for further instructions to health professionals.

## CONCLUSION

The pediatricians from the state of Amazonas who took part in the research showed a positive impact after the theoretical-practical training, improving their knowledge and skills in the neonatal diagnosis of developmental hip dysplasia.
